# A method for predicting background advertisement exposure parameters in sporting events: Televised football game approach

**DOI:** 10.1371/journal.pone.0223662

**Published:** 2019-10-17

**Authors:** Yi Xiao, Collins John, Xiaoling Ren, Pei Zhang

**Affiliations:** 1 School of Economics and Management, Shanghai University of Sport, Shanghai, China; 2 Kinesiology, Health Promotion and Recreation Department, University of North Texas, Denton, Texas, United States of America; Shandong University of Science and Technology, CHINA

## Abstract

**Background:**

The background advertisement exposure parameters (BAEP) forms a premise for sponsorship negotiation and the basis for estimating the sponsorship value of background advertising. Prediction of the BAEP has a great contribution to the sporting events organizers and sponsors in terms of negotiating, decision-making for bidding, and income-generating.

**Methods:**

Virtual Reality (VR), technology was utilized to construct a virtual three-dimensional model of the sports venue and simulate the telecast of the event. Based on VR technology and computer graphics theory, a pre-event prediction method for estimating the background advertisement exposure parameters of sporting events was put forward. The pre and post measures of the thirty BAEP of televised football games were compared to verify the effectiveness of the prediction method.

**Results:**

There was no significant difference between the pre- and post-measurement results for the same football game. The pre- and post-measurement results of the thirty BAEP of televised football games were tightly matched.

**Conclusions:**

Using the prediction method can predict the BAEP of televised football games effectively and overcomes the shortcomings of current prediction methods that inhibits the effectiveness of the prediction of exposure parameters due to changes such as the type of the sporting events, the size of the sports venue, the layout of the background advertisements, and the placement of the television cameras, etc.

## Introduction

Sports event are important components of the marketing industry. The increasing popularity of sporting events in society has led to companies resorting to marketing their products and services during sporting events to enhance their reputation and expand their markets. In the past two decades, sponsorship has become an important marketing communication tool [1–2]. There are plenty of methods to achieve marketing objectives through sponsorship apart from background images on television, such as social media, experiential hospitality, and in-store promotions [3]. However, product’s brand or logos appearing as a background advertising on television during the broadcasting of a sports event has been widely adopted by many companies due to the wide audience and almost ubiquitous distribution [4–6]. Sponsorship spending has increased tremendously during the past 30 years. According to the Worldwide sponsorship Values released by Sponsorship Research International, there was just $2 billion allocated for sponsorship worldwide in 1984 [7]. However, the number rose sharply to $65 billion in 2018 [8]. Besides, a published report showed that sports garnered 69% of total spending under the steady-state growth of sponsored activity scope [9]. In North America, Asia Pacific, Europe, Latin America, and other developed countries, the revenue from sports sponsorship has grown steadily during the past five years. Sports sponsorship has become one of the major pillar industries of their economies [10].

Normally, mega sporting events are generally associated with high operating costs and high risks in terms of return on investment [11]. For the organizers, the successful hosting of sporting events cannot be attained without the sponsors’ support, and one of the main sources of sporting events income is the revenue of sports sponsorship, which is based on the exposure of background advertisement. For the sponsors, the background advertisement exposure parameters (BAEP) is a determinant for decision-making to some extent [12]. Background advertising refers to advertisements that appear during a televised broadcast [13]. Sports venue background advertising refers to a way of advertising: the enterprises use their efforts to spread product information, drive audience consumption, and generate publicity for themselves through the grand sports event activity by sponsoring or naming it. It appears mainly in the form of billboards as the background of the game during the television broadcasting [14].

The BAEP refers to the frequency, location, and duration of the background advertisement (such as sponsor’s name, title, product, logo or brand) on the television screen during telecasting. The number of times the background advertisement image appears on the television screen is the frequency; the specific area on the television screen where the background advertisement image appears is the location, and the duration refers to the number of seconds the background advertisement image appears on the television screen [15–16]. The traditional method of measuring BAEP was to calculate the BAEP after the event, which is also known as post-event method. A number of individuals were required to manually observe and record measurement data (every background advertisement’s exposure parameters of frequency, location, and duration) from the sports videos, which was time-consuming and prone to error [17]. Since the exposure parameters measurement for each background advertisement needs to be manually counted, it leads to much-repeated viewing of the sports videos, thus creating a large workload and low efficiency [17]. With the rapid development of technology and improvements of automation, image recognition technology was developed to calculate the measurements of a video file once certain parameters (such as sponsor logos and text marks) were programmed to appear, which was time-saving and efficient [18]. The BAEP expressed as a unit of time in seconds was the premise of sponsorship negotiation, and the basis for estimating sponsorship value of the background advertisement [19–20]. In the actual measurement and application of the BAEP, the sponsorship value generated by the enterprise through the background advertisement sponsorship can be calculated by the relevant methods of advertising media science. As long as the exposure seconds and the weight coefficients for different forms of background advertising in different locations can be obtained, the median value of each background advertisement could be calculated [19–20].

In addition, the most important aspect of estimating the economic feasibility of a sporting event is the income prediction [21]. Therefore, predicting BAEP is of great significance to the sporting events organizers and sponsors in terms of negotiating, decision-making for bidding, and income-generating from a sports event.

For the sporting events organizers, the prediction of the background advertisement exposure parameters can assist them to estimate how much potential income they might obtain from the advertisement sponsors in advance, and be conducive to make sound bidding decisions whilst reduce the risks for the sponsors of a sporting event [22]. Though advertising exposure does not mean consumption, the previous study showed that there were significant differences between genders regarding the role of affective factors on the perception of advertising, which means different consumption choice would appear even for the same advertisement exposure. However, when short fixations representing unconscious processing of visual information are analyzed, no statistically significant differences between genders have been found [23]. It still contributes to the sponsor’s reasonable decision-making, agreement with the organizer of a sports event, and meanwhile reduces the transaction time and costs [24].

To ensure that a sporting event will be successfully held, it is necessary to predict the economic feasibility of the sporting event before any decision is made. Prediction is the application of scientific conjectures and judgments towards the future development of a specified object according to the object’s developing status and changing rules by drawing from the knowledge and approaches that have previously been grasped. The purpose of prediction is to provide an objective basis for decision-making or planning; thus, the premise of prediction is data [25]. For sponsorship, the premise of predicting the media value of background advertisement is to obtain its BAEP.

There are various prediction methods such as grey forecasting model, artificial neural network model, and time-series prediction method, which were widely applied in the field of finance, physical control, engineering, economics and others [26–28]. One primary method used to predict the BAEP is the time-series method, which principally uses extracted data from previous sporting events videos to predict future ones [29]. In addition, the data extracted must meet the four prerequisites: the same type of sporting events; same venue; same number and same location of background advertisements in the venue; same number and layout of television cameras [20]. Compared with traditional measurement method, the time-series method was theoretically feasible, time-saving and more efficient. The constraint of this application was that the four prerequisite conditions must be satisfied simultaneously and once changed, it would be not effective. However, in practice, it is very common that the sports venue, the number of sponsors, the setting of television cameras and background advertisements are all likely to change due to the complexity and uncertainty of each sporting event. In addition, for completely new sporting events, an accurate prediction can no longer be made by the time-series method due to the lack of historical video data. Therefore, this paper used VR technology to complete the modeling and constructing of virtual competition field, the arrangement of background advertisement (including the size and quantity of banners, the sponsor logo, and its spatial location distribution in the arena, etc.), the setting of TV broadcast position (including the number and distribution of space position of broadcast seats, the scope of responsibility of each broadcasting settings, etc.), and the simulation of sporting events television broadcasting process. Compared with the time-series method, the most significant advantage of this method was that the BAEP of different sporting events can be predicted, whether new or previously held, as long as the rules of television broadcasting had been attained.

VR technology is a technique which uses computer technology to create a virtual three-dimensional environment that consists of visual, audio, and tactile senses. By means of special input and output equipment, the users can naturally interact with the objects in the virtual three-dimensional venue and experience the “immersive” feeling. Three basic characteristics of VR technology are immersion, interaction, and imagination [30]. VR technology has been extensively used to advance the training in the aerospace industry, medicine, military, education, and architecture, as well as in product design, film and television program creation, and other fields [31–34]. VR technology has also been widely applied to various areas of sport, such as the three-dimensional simulation of human body movement, the simulation of large choreographic arrangements, the simulation of gymnastics training, weightlifting, skiing, golf, and other sporting events [35–38].

Considering the deficiencies of the current methods for predicting the BAEP and the popularity and effective use of VR technology in numerous fields, VR technology was applied to establish a pre-event prediction method for estimating the background advertisement exposure parameters of a future football game. In addition, the theoretical framework and basic steps of this method were also presented. VR technology was used to construct a virtual three-dimensional model of the sports venue and simulate the telecast of a sports event to calculate the background advertisement exposure parameters automatically. It was designed to provide an effective and quantitative method for predicting. The result would assist in the decision-making process relating to bidding and sponsorship negotiation, and the method overcomes the shortcomings of current prediction methods that inhabited the effectiveness of the prediction due to the changes in the type of the sporting events, the size of the sports venue, the layout of the background advertisements, and the placement of the television cameras.

## Methods

### Prerequisite conditions for predicting BAEP

There are many factors that may influence the exposure of background advertisement. However, in this study the following five factors were considered to be the prerequisite conditions for predicting BAEP: the type of sport events, the size of the sport venue, the location of the background advertisements in the stadium, the setting of the television cameras (including the quantity and the location of each camera), and the rule of television broadcasting (the frequency and duration of each camera used in the television broadcast).

Although the television broadcasting of sports events differs by the type of sport, venue, and television broadcasting team, etc., the television broadcast will still follow certain rules [20]. Generally, if the sports venue is changed, the rule of television broadcasting will also change. However, for a specific sporting event, the size of the venue, the number of background advertisements and television cameras, etc. may change, the size of the competition court/field is basically unchanged, and the TV broadcasting mainly focuses on the competition court/field. Furthermore, the number and the layout of the host and auxiliary television cameras and the duty scope of each camera would be relatively fixed. In addition, the TV broadcasting teams have summed up the corresponding broadcasting rules for different sizes of venues (large, medium and small) and different types of sports events based on years of experience and empirical knowledge in broadcasting sports events [39].

In this study, the mathematically derived method was applied to extract the statistical rule of television broadcasting from 30 previous football game videos that met the other four prerequisite conditions. The rule of television broadcasting drawn from previous occasions was used to predict the BAEP for new sporting events.

### Virtual prediction algorithm

In this study, the virtual prediction algorithm was the key component of the prediction of BAEP. Its main task was to automatically generate the exposure of background advertisement and calculate the exposure parameters (frequency, location, and duration) for each exposure on the virtual television screen. The virtual prediction algorithm was designed based on the three-dimensional projection transformation theory [40], and the seed-filling algorithm of computer graphics, which can automatically complete the calculation of background advertisement exposure parameters [41–43].

#### Generation of background advertisement exposure

Background advertisements located in the sports stadium are projected onto the TV screen to be exposed during television broadcasting. From the perspective of computer graphics, the process of background advertisement exposure is essentially a process of transforming a three-dimensional object in a stadium into a two-dimensional object and displaying it on a television screen through a series of three-dimensional image transformations [44]. Using the three-dimensional projection transformation theory of computer graphics, a three-dimensional coordinate point P_3D in the stadium can be converted into a two-dimensional screen coordinate point P_Screen, namely: P_Screen = P_3D*Matrix, where P_Screen is the screen coordinate point, P_3D is the spatial coordinate point, and Matrix is the projection transformation matrix (it was obtained by the setting of television cameras in this paper) [44].

**Fig 1:**
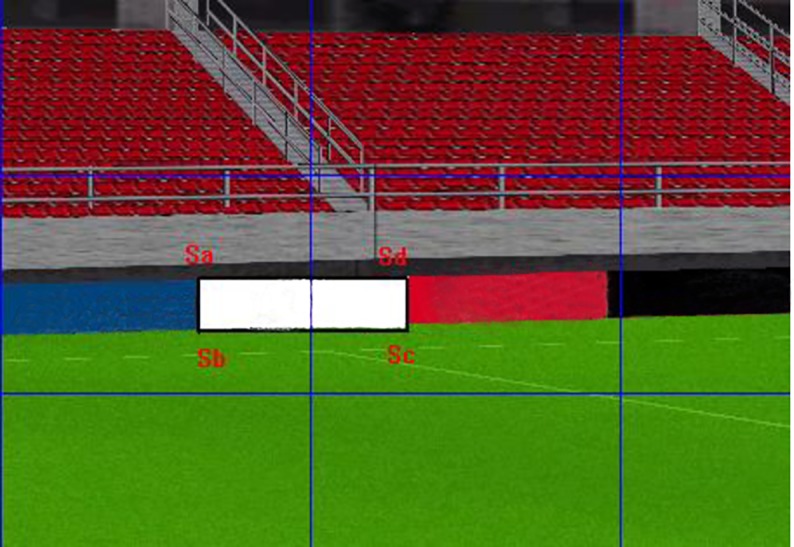
Virtual exposure of background advertisement.

In the virtual three-dimensional sports stadium, if we selected four spatial vertices A, B, C, and D representing a billboard boundary, the corresponding two-dimensional screen coordinate points Sa, Sb, Sc, Sd of the four spatial points A, B, C, and D would be obtained through three-dimensional projection transformations. By connecting the four points of Sa, Sb, Sc, and Sd in turn, a quadrangle was obtained, which was the background advertisement that television viewers saw on the virtual TV screen [44], as shown in Fig 1.

#### Seed-filling algorithm of virtual background advertisement exposure

**Fig 2:**
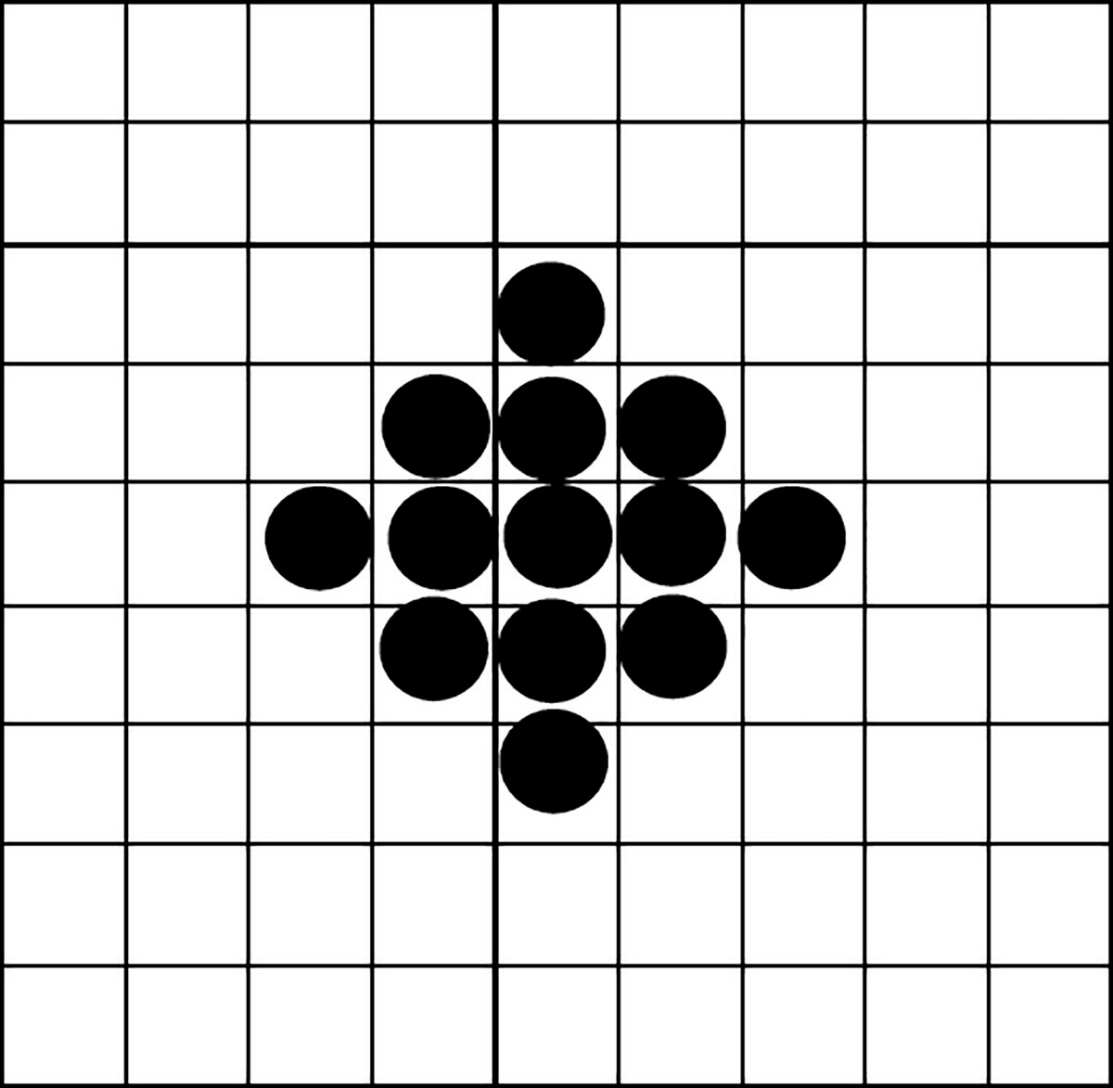
The pixel filling of background advertisement exposure.

Using the seed-filling algorithm of computer graphics [43], the distribution of the quadrangle SaSbScSd representing the background billboard on the virtual TV screen can be drawn. Dividing the number of pixels within the quadrangle by the total number of pixels on the TV screen (pixel width * pixel height of the screen) yields the ratio of background advertisement to screen area, as shown in Fig 2. In order to specifically describe the exposure position of each background advertisement on the television screen, the television screen was divided into a 4-by-4 grid with 16 cells.

#### Recognition of background advertisement

The BAEP is mainly related to the size and specific location of each background billboard in the stadium and has no direct relationship with the specific content (brand or logo) of each background advertisement. Therefore, in this study, the recognition of different background advertisement’s digital image was replaced with the recognition of each background advertisement’s location in the sports stadium, which improved the efficiency and accuracy of the recognition of background advertisements [39].

#### Calculation of BAEP

The BAEP includes three parameters, which are position, duration, and frequency, separately. The position of background billboard on the television screen was obtained by the distribution of the pixel points of the quadrangle SaSbScSd on the virtual television screen. The duration of each background advertisement exposure was automatically calculated by the system. Every measured exposure parameter data were automatically saved to the result file (an excel file). The total frequency can be obtained by summarizing the detailed data of each exposure. At this point, the 3 exposure parameters (frequency, duration, and position) of all background advertisements were calculated during the TV broadcasting of the entire game.

The pseudocode code for the proposed virtual prediction algorithm is shown as following:

input: N        /* total number of broadcasting cameras */

        the rule of TV broadcasting

output: BAEP        /* background advertisement exposure parameter(excel file) */

Initialization:

    N = 1

    For i = 1 to N

        Calculating the BAEP

end

**Fig 3:**
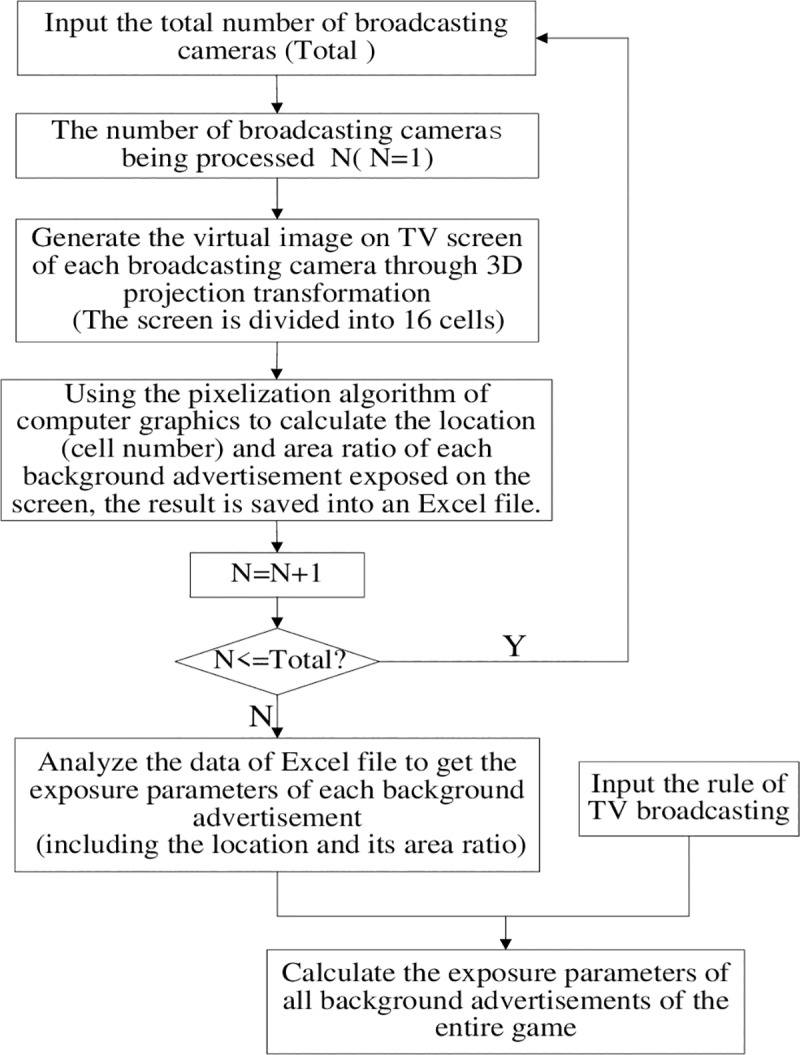
The flowchart of the calculation of BAEP.

The specific flowchart was shown in Fig 3.

#### The steps of the virtual prediction method

**Fig 4:**
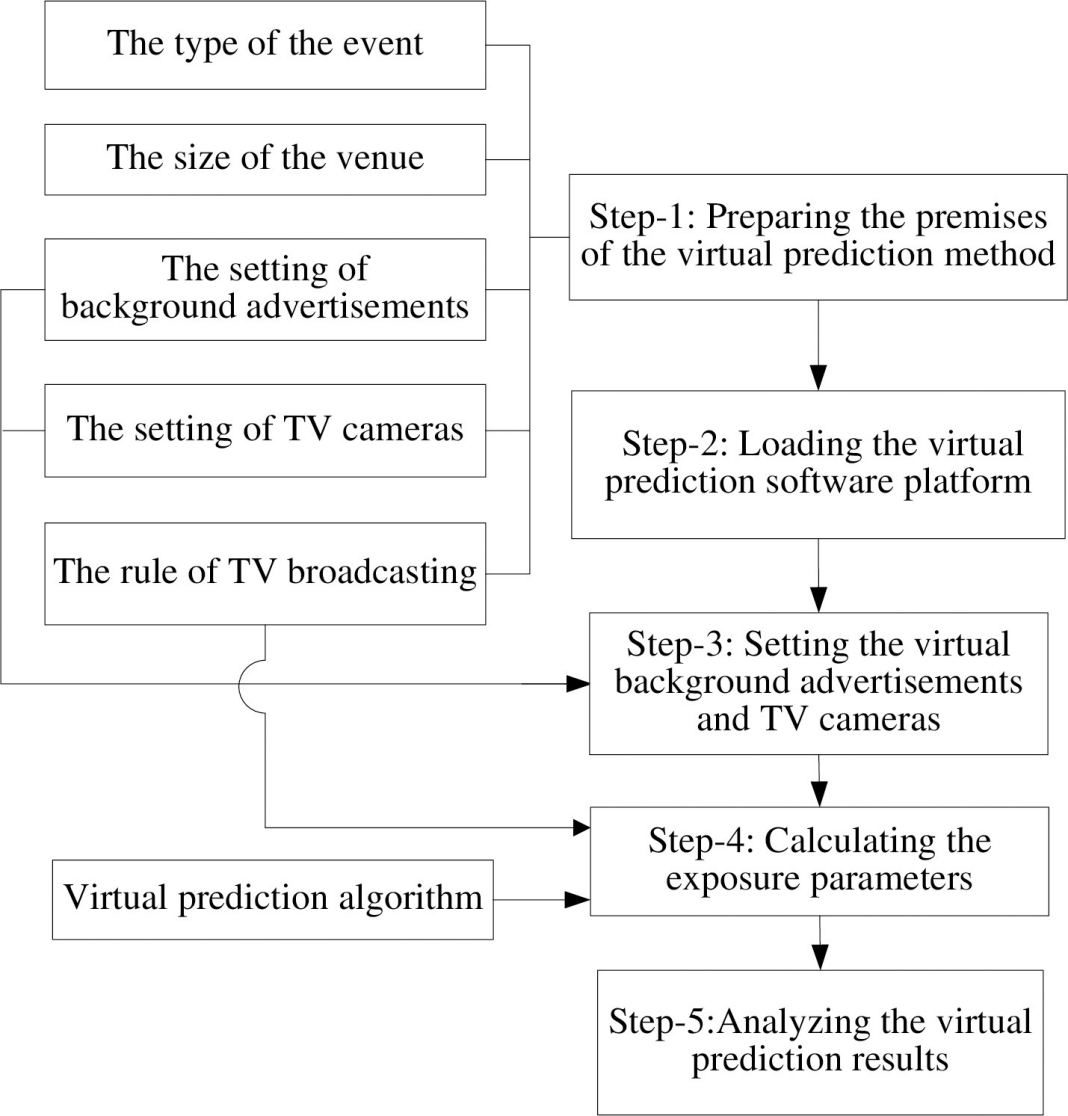
The steps of the virtual prediction method.

The basic step of the virtual prediction method was a 5-step process. It incorporated the basic procedures of the post-event method (manually observing and recording measurements) that was used to extract the rule of a television broadcast. A virtual prediction software platform was constructed. The software can simulate the actual game and television broadcasting scenario, and measure the background advertisement exposure parameters automatically based on a virtual prediction algorithm. The steps of the virtual prediction method were presented in Fig 4.

Step-1: Prepare the premises of the virtual prediction method. According to the post-event method, an intensive analysis of previous sport videos, meeting the four prerequisite conditions, was conducted to establish the premises of the virtual prediction method, such as the type of sport events, the size of venue, the number and locations of background advertisements, the number and the layout of television cameras, and the rule of television broadcast. The rule of television broadcast was the most direct and major premise related to the BAEP and was mathematically derived from the results obtained by the post-event measurement method.

Step-2: Loading the virtual prediction software platform. According to the premises of virtual prediction obtained in Step-1, the virtual prediction model included: the virtual three-dimensional stadium model, the playing field or court model, the virtual background advertisement model, the virtual television camera model, the input (the rule of television broadcast), processing of the input, and the output (the BAEP).

The software platform used for virtual prediction was developed to automatically perform the virtual prediction of BAEP. Its five main functions were: loading the virtual prediction model, arranging the virtual background advertisements and television cameras, importing the statistical rule of a television broadcast, simulating the television broadcasting and automatically calculating the BAEP according to the virtual prediction algorithm.

Step-3: Setting the virtual background advertisements and television cameras. According to the analysis of the previous sports videos of an event, the venue, the background advertisements, the television cameras, and the playing field were loaded and arranged in the virtual three-dimensional prediction platform, which fulfills the preparation for the premises of the virtual prediction method.

Step-4: Calculating the exposure parameters. After all the other procedures had been completed, the rule of television broadcast was imported into the virtual three-dimensional prediction software platform, and the BAEP was automatically calculated according to the virtual prediction algorithm during the simulated television broadcast.

Step-5: Analyzing the virtual prediction results. Data normality was verified by using the Kolmogorov-Smirnov test. Pearson’s Product Moment Correlation Coefficient was conducted to examine the association between the virtual prediction results and the post-event measurement results. A t-test was performed to determine the difference of the average exposure time between the pre- and post-event measurement results. The prediction model was cross-validated with a repeated random sampling approach. Statistical significance was defined at 5% (*p* < 0.05).

## Results

### The premises of virtual prediction

The 30 football games played at the Shanghai Hongkou Football Stadium in 2016 were used to extract the information for determining the five premises related to the virtual prediction method of BAEP.

#### The rule of the television broadcast

For Chinese Football Super League, there are total 13 television cameras used for the TV broadcasting in Shanghai Hongkou Football Stadium. Generally, each camera has a clear responsibility scope for the television broadcast [39], which determines the spatial location distribution of each camera in the arena. Different cameras have different responsibilities scope for TV broadcasting. Some are responsible for penalty kicks, some are responsible for the shooting, while others are responsible for panoramic views and so on. Furthermore, the frequency and duration of each camera used during the television broadcast are different. The rule of television broadcast based on the 30 football game videos was shown in Table 1 which revealed the amount of broadcasting time, in seconds, each television camera was used during the whole football game. In this study, camera no.1, located at a higher point in the middle of the stadium, is the host camera and has the highest broadcasting time. The second is camera no.2 and no.3, which are also located close to the center of the stadium. Camera no.13 is an auxiliary one, whose duty scope is for panoramic views, and has the lowest broadcasting time.

**Table 1:** The rule of television broadcast.

Television Camera No	Time Length (seconds)	Television Camera No	Time Length (seconds)
1	3201	8	21
2	829	9	105
3	451	10	4
4	226	11	14
5 6 7	324 92 261	12 13	131 3

#### The virtual prediction software platform

**Fig 5:**
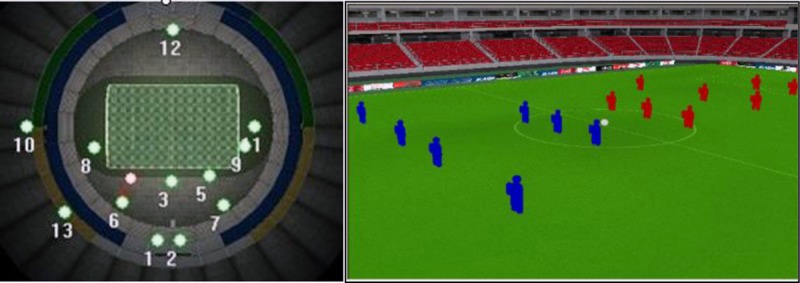
The main interface of the virtual prediction software platform.

According to the information obtained from the 30 football game videos, the virtual three-dimensional football stadium model, the virtual playing field, the virtual background advertisements, and the locations of the 13 virtual television cameras were created in the virtual prediction software platform. The main interface of the virtual prediction software platform was indicated in Fig 5.

The main interface of the virtual prediction software platform is divided into the left and right parts. On the left is the top view of the stadium, showing the distribution of the 13 camera positions in the stadium. On the right are the virtual three-dimensional stadium and virtual game scene. The duty scope of the camera for broadcasting and the exposure of background advertisements on the screen can be observed by clicking on one of the cameras in the interface on the left. In the software platform, the setting of virtual background advertisements and television cameras (including the number and spatial location) in the stadium can be completed and adjusted, which satisfies the changed prerequisites of the new sporting event.

#### The layout of background advertisement

**Fig 6:**
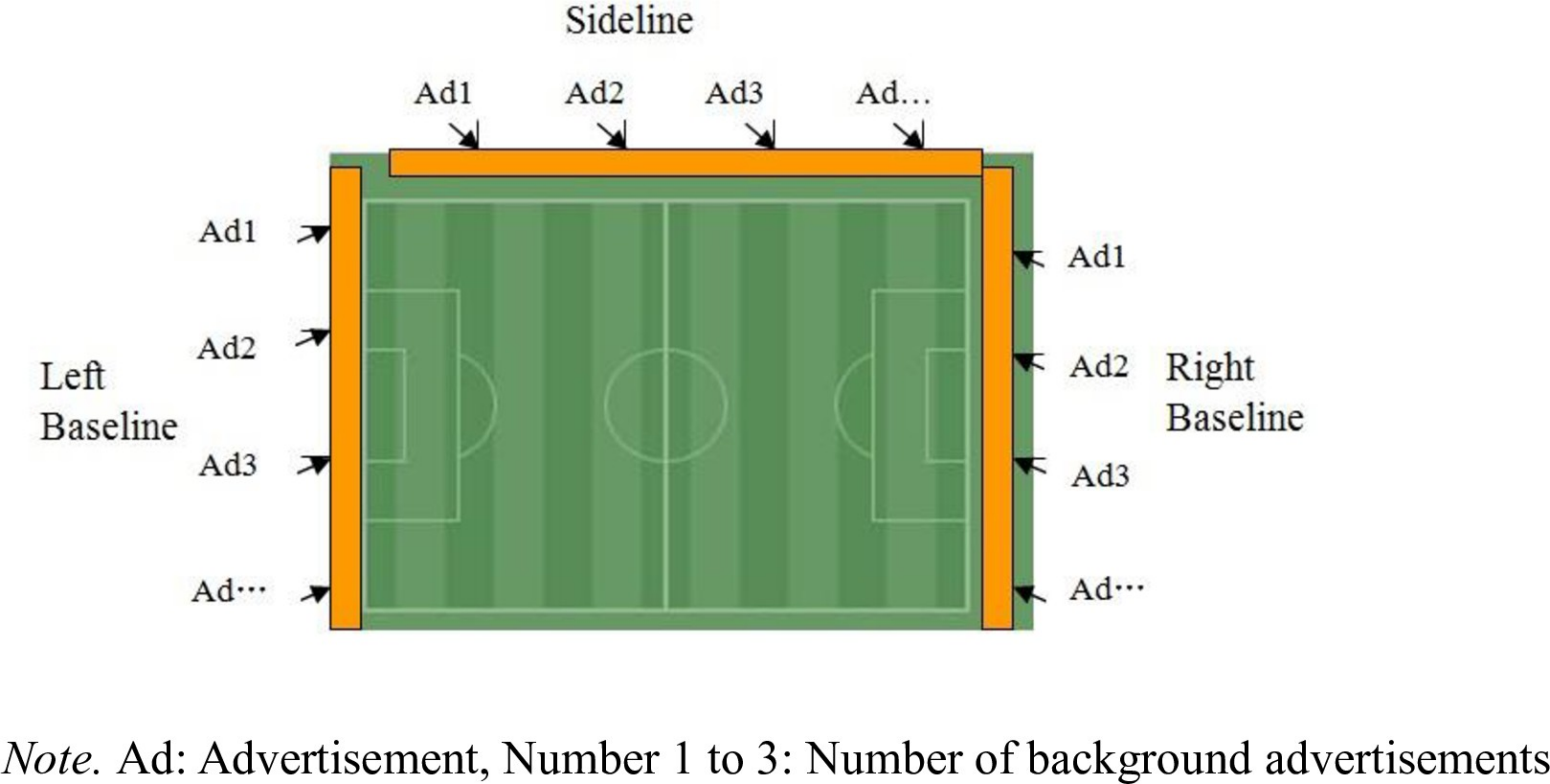
The layout diagram of background advertisement.

The BAEP is related to the specific size and location of each background banner in the stadium. For football, the background banners can be allocated on the three sides of the playing field (the sideline, the left baseline, and the right baseline. The quantity of background banners depends on the size of the football field and the size of the background banner. In this study, there were seven background advertisements located across the sideline and four background advertisements placed at each baseline, all of which generated a total of 15 advertisement locations. The background banners were numbered in the left-to-right, top-to-down direction. The location diagram of background advertisements in the virtual three-dimensional football stadium was shown in Fig 6.

#### The predicted results of BAEP on the 16-screen cell

**Fig 7:**
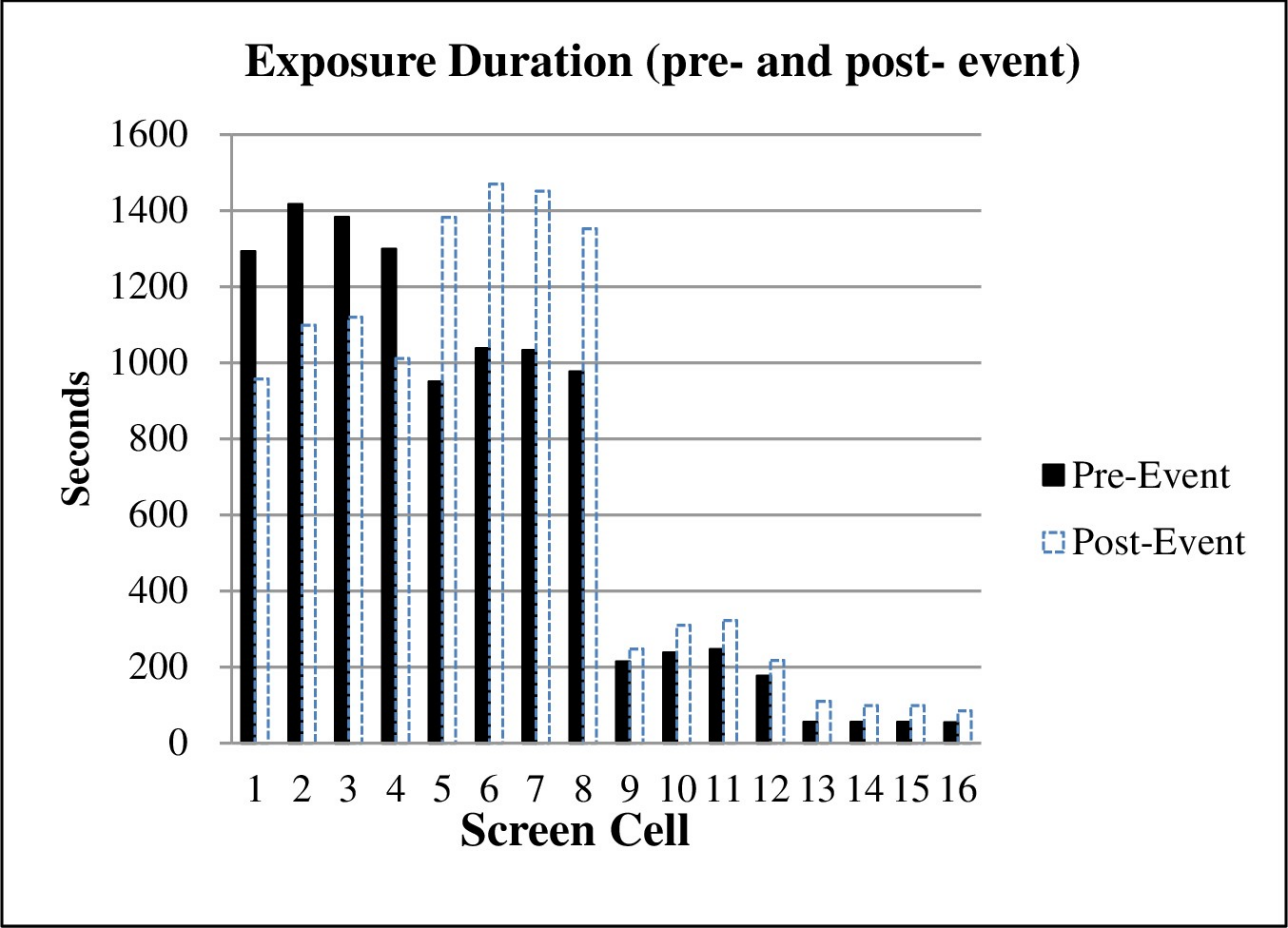
The pre- and post-measurement results of BAEP.

After the rule of television broadcasting was imported into the virtual three-dimensional prediction software platform, the BAEP on the 16-screen cell was automatically predicted according to the virtual prediction algorithm. The prediction (pre-event) results are shown in Fig 7, which indicated that the largest duration of background advertisement exposure occurred in cells 1 to 4, with approximately 5388 seconds of exposure. This was closely followed by cells 5 to 8 which accounted for approximately 3993 seconds of background exposure. Cells 9 to 16 had a lower number of seconds of background exposure than Cells 1 to 8. Cells 9 to 12 accounted for approximately 872 seconds of exposure, and Cells 13 to 16 accounted for the lowest duration of the exposure, with approximately 215 seconds of background exposure.

#### The post-measurement results of BAEP on 16-screen cell

The BAEP for each of the 30 Hongkou football games was measured after the game was held (post-event) by using the manually observing and recording method. The measurement results of the BAEP for the 30 football games was calculated by summarizing the duration of exposure for each of the 16 screen cells separately. The results were then divided by 30 to get the averaged results (Fig 7). As Fig 7 indicated, the largest duration of background advertising exposure occurred in cells 5 to 8, with approximately 5654 seconds of exposure for these four cells. This was closely followed by cells 1 to 4, which accounted for approximately 4186 seconds of background exposure. Cells 9 to 16 had lower seconds of background exposure than Cells 1 through 8. Cells 9 to12 accounted for approximately 1096 seconds of exposure, and Cells 13 to 16 accounted for the lowest duration of exposure, with approximately 389 seconds of background exposure.

#### The comparison of pre- and post-measurement results of BAEP on 16-screen cell

The post-event measurement results of BAEP were generally used to represent the actual background exposure. To verify the effectiveness of the prediction method, the predicted results were compared with the post-event results (Fig 7). As Fig 7 indicated, the exposure duration of screen cells no.1-4 were systematically overestimated and no.5-8 were underestimated compared with the post-event observed values. The reason may be that during the real-time TV broadcasting of football matches, players usually cover parts of the background banners, especially in the center of the TV screen, which will affect the post-event observed BAEP, but has little influence on the predicted results.

The Kolmogorov-Smirnov test result showed that the data of pre- and post-measurement results of BAEP on 16-screen cell were normally distributed (*p* = 0.197, *p* = 0.253, respectively). There was no significant difference between the post-measurement results (M = 707.81, SD = 361.49) and the prediction results (M = 654.31, SD = 354.83) of BAEP on all 16 screen cells [t = -0.816 (15), *p* = 0.428]. There was significant correlation between the pre- and post-measurement results of BAEP on 16-screen cell (r = 0.890, *p* < 0.001). The prediction model was cross-validated using ANOVA calculations for the lack-of-fit test (F = 0.680, *p* = 0.650).

The prediction error was calculated by using the difference between the average of predicted BAEP and the average of observed BAEP divided by the average of observed BAEP. For screen cells no.1-4, no.5-8, no.1-8, and no.1-16, the prediction errors were 20.3%, 24.7%, 4.6%, and 7.4%, respectively. For screen cells no.1-4 and no.5-8, the prediction errors were both over 20%. However, for screen cells no.1-8 and no.1-16, the prediction errors were both lower than 8%. The conclusion was that the overall prediction errors are acceptable. Furthermore, the prediction error of this virtual prediction method was tested several times and it was found that the overall prediction error of screen cells no.1-8 and no.1-16 were also both lower than 10%. The analysis indicated that although the shapes of the columnar curves of the two histograms were not exactly matched, the curved shapes displayed similar features, which meant that while there was a certain amount of error between the two measurements results, the predicted results were closely matched the post-event results and were deemed credible to some degree.

#### The comparison of pre- and post-event exposure time of advertisement location

In this study, there were seven background advertisements located across the sideline, and four background advertisements placed at each baseline (Fig 6).

### The predicted exposure time for different advertisement locations

**Table 2** indicated the amount of exposure-time predicted for each of the 15 background advertisement locations during a football game. The left baseline location #1 received 871 seconds of exposure time during television broadcasting, which was the highest. The results also showed that six of the seven sideline locations had the most exposure time with location #2 receiving 621 seconds and location #5 having the highest exposure time with 773 seconds during the entire television broadcasting. However, the four advertising locations along the right baseline all had the lowest amount of exposure time with the right baseline location #3 only receiving 99 seconds of exposure time.

**Table 2:** The post- and pre-event exposure time of advertisement location.

Advertisement Location	Post-event Time (seconds)	Pre-event Time (seconds)	Difference (seconds)
S-1	387	338	49
S-2	502	621	119
S-3	517	632	115
S-4	571	651	80
S-5	858	773	85
S-6	665	636	29
S-7	588	633	45
R-B-1	209	180	29
R-B-2	240	282	42
R-B-3	142	99	43
R-B-4	30	101	71
L-B-1	966	871	95
L-B-2	655	576	79
L-B-3	485	429	56
L-B-4	362	330	32

*Note*. S: Sideline, R-B: Right Baseline, L-B: Left Baseline, Number 1 to 7: Location of background advertisements.

### The post-event exposure time for different advertisement locations

The post-event exposure time of the 15 advertisement locations for each of the 30 football games was measured and the average exposure time of the 30 football games was calculated. The results were also presented in Table 2. The left baseline location #1 received the greatest amount of television broadcasting exposure time with 966 seconds. The left baseline location #2 received the fourth-highest amount of television broadcasting exposure time with 655 seconds. Similar to the pre-event prediction, the sideline post-event locations were all in the top 10 locations with location #5 receiving the highest sideline exposure time with 858 seconds. Similar to the pre-event method, the four right baseline advertising locations received the lowest amount of television broadcasting exposure time with location #4 only receiving 30 seconds for the duration of the entire game’s television broadcasting.

### Comparison of pre- and post-event exposure time of advertisement location

The Kolmogorov-Smirnov test result showed that the data of pre- and post-event exposure time of advertisement location were normally distributed (*p* = 0.993, *p* = 0.642, respectively). Pearson’s Correlation coefficient revealed that the correlation between the pre- and the post-event exposure time of advertisement location was significant (r = 0.959, *p* < 0.001).

To examine the “fitness” of the pre-event data to the post-event data, a t-test was conducted to determine whether the average exposure time, in seconds, across all 15 advertising locations was significantly different between the pre- and post-event data. The result showed that there was no significant difference between the post-event exposure time (M = 476.80, SD = 243.64) and the pre-event exposure time (M = 478.47, SD = 258.57) of all 15 advertisement locations [t = .088 (14), p = 0.931], which meant that the average exposure time of advertisement location generated by the two methods produced similar results.

The prediction model was also cross-validated using ANOVA calculations for the lack-of-fit test (F = 0.355, *p* = 0.835). The *p-*value was over 0.05, which indicated that the prediction of the exposure time of advertisement location was effective.

## Discussion

Due to the uncertainty inherent characteristics of the football event, television broadcast, and the complexity of the virtual predictive measurement of the BAEP, some errors were inevitable between the predicted results and the actual post-event measured results. Further examination indicated that there were some potential factors that might have contributed to errors detected between the virtual prediction method and the post-event method.

The focal length is controlled by the television broadcasting company during the actual game. Consequently, the angle of the camera, the target area, and associated focal length may change during the game, all of which were decided by the broadcast company. Predicting these changes by using the existing technology is difficult. For this study, the shooting angle and focal length of each camera were set statically, which might have led to some differences between the actual and predicted game preconditions. Another potential source of error is built on the statistical rule of television broadcasting. It is difficult to predict actual situations during a football game due to television broadcasting’s uncertainty and randomness, thus the rule of television broadcasting might lead to certain deviations with the actual television broadcast. The other source of potential error is the setting of the television cameras. Although each camera generally has a clear responsibility for the television broadcast, some artificial errors in setting the television cameras in the virtual prediction platform are still inevitable, which will lead to some errors in the predicted results.

Although the media value of background advertisement can be calculated by obtaining different types of advertisement duration and corresponding weight coefficients, the value obtained by the measurement formula is only a theoretical value rather than an actual one. There is no doubt that the higher the BAEP, the higher the theoretical media value, even if there were deviations from the actual media value. In addition, the results of the post-event measurement method, whose process was laborious and error-prone, have generally been used to represent the actual background exposure. This is also a factor that cannot be ignored in the occurrence of errors.

## Conclusion

The virtual prediction of the BAEP of a sports event involves multi-disciplinary approaches, such as prediction theory, three-dimensional modeling theory, VR technology, software development, and database management. Virtual prediction method overcomes the shortcomings of the existing prediction methods, such as the prediction cannot be done effectively due to the changes in the type of sports events, the size of sports venue, the layout of background advertisements, and the placement of television cameras. Due to the complexity, uniqueness, and uncertainty of each sporting events, the sports venue, the number of sponsors, the setting of television cameras and background advertisements are all likely to change. When changes occurred, it is very difficult to find enough sports videos which meet the latest changed prerequisite conditions, which renders the existing predicting method ineffective.

These limitations can be overcome when the virtual prediction method is utilized because it can construct a three-dimensional prediction model of a sport venue based on the five prerequisite conditions (the type of sport events, the size of sports venue, the layout of background advertisements, the placement of television cameras, and the rule of television broadcasting) and develop a software platform for the virtual prediction of BAEP. Once prerequisites changed, VR technology would be used to set up a new virtual sports venue according to the changed prerequisite conditions, and complete the setting of virtual background advertisements and the television cameras in the virtual three-dimensional model, which satisfies the changed prerequisites of the new sporting events. In addition, the versatility of this prediction method can be expanded across other types of sports events such as tennis, basketball and so on once the prerequisite conditions had been obtained.

## Implication

The virtual prediction of the BAEP has much practical and theoretical value. This study utilized the VR technology in an attempt to predict the BAEP and avoided the deficiencies of the existing prediction methods. It also provided a new and effective technical method for predicting the BAEP of football event, which broadens the application field of VR technology and further promotes the application research of this technology. Furthermore, this study advanced the theoretical procedures that used the virtual prediction method to predict the BAEP for football events. In order to provide a more reliable foundation for its practical application and to verify the effectiveness of its algorithm and process, it was applied to predict the BAEP of a football game.

The virtual prediction method can identify potential background advertisement locations which have high exposure values in terms of frequency and duration of exposure. In the virtual prediction software platform, the background advertisements can be relocated and the BAEP for different location layouts can be calculated. The background advertisement locations that have higher exposure value can then be identified from the measurement results, which provides the event organizers and sponsors a valuable reference for the background advertisements’ layout plan.

The virtual prediction software platform helps to improve the television broadcast and maximize the interests of television media by adjusting the length of time each television camera used in the television broadcast, and the BAEP can be measured related to this adjustment. The ratio of each camera’s change in broadcasting time to the change in total exposure time for all background advertisements can be calculated, which helps the television broadcast company identify the deficiencies of the television broadcast so as to improve the game’s television broadcasting and maximize the total exposure time-length of background advertisements. Meanwhile, it would also maximize the interests of television media.

In addition, the successful application of the virtual prediction method would have great significance to sponsorship negotiation between the event organizer and sponsors. The event organizers can estimate the how much potential income they might obtain from the sponsor before the sporting events; the sponsors can know in advance how much time, in seconds, their advertisements will appear on the television screen during the game, which can prompt both sides to reach a consensus and reduce the cost of sponsorship negotiation.

## Limitations

From the sponsorship perspective, sponsorship exposure is not the only metrics to measure ROI in sport sponsorship and the exposure does not necessarily imply the audience’s attention. There are many other factors that affect the effectiveness of sponsorship, such as brand sponsor-team congruence, brand confusion and attention to advertising diminished by the excitement of the even [45–46]. In this paper, the BAEP is mainly affected by the size and specific location of each background billboard in the stadium and have no direct relationship with the specific content (brand or logo) of each background advertisement. So there was no discussion on factors as color consistency, brand confusion, and other specific issues, which may affect the effectiveness of sponsorship. In addition, the types of sponsoring (commercial sponsorships or philanthropic sponsorships) have an impact on sponsors’ brand equity, thus affect investors’ confidence for sponsor [47]. However, these factors were of great significance to sponsorship effectiveness and should be further discussed in the later study.

For new sporting events, the lack of historical video data will decrease the accuracy of the results predicted by this method. Since this study focused on the prediction (pre-event) method for determining the BAEP, it was highly recommended that the information extracted from the post-event analysis of future football games be incorporated into the prediction model. This recommendation is based on the assumption that the greater the number of post-sport video analysis that can be conducted, the more refined the prediction model and the more accurate the predicted BAEP.

## Supporting information

S1 Dataset The pre- and post-measurement results of BAEP. (SAV)
